# The relationship between adverse childhood experiences and depression and anxiety in adolescents: the chain mediating effect of perceived stress and self-hate

**DOI:** 10.3389/fpsyg.2026.1762403

**Published:** 2026-04-29

**Authors:** Bingjie Zhou, Meng Qi, Yuejiao Yu, Songying Fang, Shuangjiang Zhou, Leilei Wang, Jingxu Chen

**Affiliations:** 1 Department of Psychology, Chengde Medical University, Chengde, China; 2 Beijing Huilongguan Hospital, Capital Medical University, Beijing, China; 3 Qingdao Eighth People’s Hospital, Qingdao, China

**Keywords:** adolescence, adverse childhood experiences, depression and anxiety, perceived stress, self-hate

## Abstract

**Objective:**

Adolescent emotional issues have gained increasing global attention. The connection between Adverse Childhood Experiences (ACEs) and emotional issues in adolescents is well-established. However, the specific psychological pathways that explain this link are not yet fully understood. Our study aims to clarify the link between ACEs and depressive/anxious symptoms in a large Chinese adolescent cohort.

**Methods:**

We conducted a cross-sectional analysis with 7,158 students from Shandong Province, China, using a stratified cluster sampling method. Participants completed a series of self-report scales: the Adverse Childhood Experiences Scale (ACE-R), the Perceived Stress Scale (PSS-10), the Self-hate Scale (SHS), and the Patient Health Questionnaire-4 (PHQ-4). To test our chain mediation hypothesis, we used correlation analyses and a bootstrap approach.

**Results:**

Female adolescents and those with poorer academic performance had higher scores on childhood adverse experiences, perceived stress, self-hate, and emotional symptoms (*p* < 0.05). High school students scored higher than middle school students in terms of childhood adverse experiences and self-hate (*p* < 0.05). Only children had higher scores on childhood adverse experiences compared to non-only children (*p* < 0.05). Perceived stress and self-hate serve as chain mediators in the relationship between childhood adverse experiences and depressive-anxiety symptoms in adolescents.

**Discussion:**

This study expands the research on the relationship between adverse childhood experiences and depressive and anxious emotions in adolescence, providing new insights for reducing the negative impacts of depressive and anxious emotions in adolescents and promoting their mental health.

## 1 Introduction

In recent years, mental health problems among adolescents have become increasingly prevalent, with anxiety and depression emerging as the primary manifestations. These conditions contribute significantly to the global mental health burden, ranking within the top 25% of disabling factors worldwide (Tan et al., 2024). Adolescence is a critical period marked by profound biological, psychological, and social changes (Blakemore, 2019). The high incidence of depression and anxiety during this stage significantly threatens individuals’ physical, mental, and social well-being (Thapar et al., 2022).

Adverse Childhood Experiences (ACEs) have been consistently identified as a strong predictor of emotional problems in adolescence. ACEs refer to events that occur during childhood or adolescence and have the potential to negatively impact an individual’s mental and physical health (Russell et al., 2025). Numerous studies have shown that ACEs significantly increase the risk of developing depression and anxiety, making individuals more susceptible to these disorders during adolescence (Song et al., 2024; Hughes et al., 2017). However, it remains unclear whether ACEs directly cause emotional problems in adolescents.

Recent research suggests that the relationship between ACEs and emotional problems may be mediated by perceived stress. Perceived stress refers to the psychological experience of stress in response to events that are perceived as uncontrollable or difficult to resolve (Xu et al., 2024). According to the “stress sensitization” theory, early adverse experiences may make individuals more sensitive to stressors later in life (McLaughlin et al., 2010). This heightened sensitivity to stress can lead to increased levels of stress, which, over time, may precipitate depression and anxiety (Grant et al., 2014).

Furthermore, self-hate, a negative self-evaluation, may also play a critical role in this process. Self-hate refers to the persistent denial and hostility toward one’s own value, abilities, or appearance—a form of negative emotional self-regulation (Liu et al., 2023). Studies have shown that self-hate is a risk factor in cognitive models of depression and anxiety and can directly contribute to the onset of emotional symptoms (Gilbert et al., 2010).

Importantly, perceived stress and self-hate may not operate in isolation; rather, they are interrelated. Individuals experiencing high levels of perceived stress may reinforce negative self-evaluations through repeated stress encounters, which in turn exacerbates self-hate and emotional symptoms such as depression and anxiety (Kotera et al., 2021). Thus, a potential mediating chain could exist, wherein ACEs lead to increased perceived stress, which subsequently fosters self-hate, ultimately contributing to depression and anxiety.

While these mechanisms are well-documented in adults, their manifestation in adolescents remains a critical research gap. Meta-analytic evidence indicates that negative self-schemas link early maltreatment to chronic depression and stress sensitivity in adults (Hughes et al., 2017). Adolescence represents a unique developmental window where the prefrontal cortex remains immature, rendering adolescents more vulnerable to social-evaluative stress (Blakemore, 2019). According to Stress Sensitization Theory, early exposure to ACEs can prime the developing neuroendocrine system. When later confronted with social-evaluative stress, these sensitized individuals exhibit exaggerated physiological reactivity, including hyper-activation of the hypothalamic–pituitary–adrenal (HPA) axis, characterized by altered cortisol secretion and heightened autonomic nervous system responses (Kuhlman et al., 2021). Therefore, investigating these psychological pathways in adolescents is essential for developing targeted early interventions.

This study integrates Stress Sensitization Theory with Cognitive Models of Depression to explain the underlying mechanisms. According to the stress sensitization framework, early exposure to ACEs can lead to neurobiological dysregulation, lowering the threshold for stress reactivity in later life. This mechanism explains the path from ACEs to elevated Perceived Stress. From a cognitive perspective, persistent high stress may catalyze negative self-referential processing. When adolescents perceive environmental demands as uncontrollable, they may internalize these setbacks, leading to Self-hate—a stable negative self-evaluation and a direct risk factor for emotional disorders. By synthesizing these views, we propose a comprehensive psychological pathway where ACEs influence depressive and anxiety symptoms through the interplay of perceived stress and self-hate.

## 2 Materials and methods

### 2.1 Participants

This research employed a cross-sectional observational design. We used a stratified cluster sampling method to recruit participants from middle and high schools in Shandong Province, China. The participants were aged 12–18 years, with an overall mean age of 15.82 years (SD = 1.64). To ensure sample representativeness, we stratified schools based on geographical region (urban vs. rural) and educational level. The junior high school cluster (*n* = 3,579) had ages ranging from 12 to 15 years. The senior high school cluster ages ranged from 15 to 18 years. A total of 7,734 questionnaires were returned. Incomplete responses, erroneous entries, and questionnaires with excessively short completion times were excluded, leaving 7,158 valid responses. The effective response rate was 92.56%. Prior to distributing the questionnaires, the administrators underwent specialized training, and respondents were instructed to complete the questionnaires independently and in strict accordance with the guidelines. School teachers supervised the completion process. The study was approved by the Ethics Committee of Beijing Hui Long Guan Hospital (Approval No. 2021-17-Department), and informed consent was obtained from all participants and their guardians.

### 2.2 Measures

#### 2.2.1 Demographic measurement

A self-designed questionnaire was used to collect demographic information, including age, gender, grade level (high school/middle school), whether the participant was an only child (yes/no), and academic performance (poor/average/excellent).

#### 2.2.2 Assessment of adverse childhood experiences

The Adverse Childhood Experiences Scale (ACE-R) (Wang et al., 2018) is a unidimensional scale used to measure adverse experiences before the age of 18. It contains 14 items, each representing a specific type of adverse event. A score of “0” is assigned if the event did not occur, and “1” if it did. The total score is obtained by summing the scores for all 14 items, with a maximum possible score of 14. A total score of ≥1 indicates the presence of adverse childhood experiences, with higher scores reflecting a greater number of traumatic events. The Chinese version of the ACE-R has been verified to have good content validity and structural validity in Chinese adolescent samples, with the Cronbach’s *α* coefficient ranging from 0.82 to 0.88 in previous studies (Wang et al., 2018). In this study, the Cronbach’s α coefficient for the ACE-R was 0.85.

#### 2.2.3 Assessment of perceived stress

Perceived stress was assessed using the Perceived Stress Scale (PSS). The copyright for this scale was obtained from ePROVIDE, a platform provided by the MAMI Research Trust (https://eprovide.mapi-trust.org/; ID: SPECIAL TERMS No113766). The PSS is one of the most widely used tools for measuring individuals’ perceived stress levels in both home and external environments. The scale includes two dimensions: one measures the current perceived stress level (PSS-Stress Perception), and the other evaluates the individual’s coping capacity (PSS-Coping Capacity). In this study, the 10-item short version of the PSS (PSS-10) was used, with scores ranging from 0 to 4 on a Likert scale. The total score ranges from 0 to 40, with higher scores indicating greater perceived stress. The Chinese version of the PSS-10 has shown good reliability and validity. Its Cronbach’s *α* coefficient was 0.80 ~ 0.85 in previous relevant studies (Sun et al., 2019; Xu et al., 2024). In this study, the Cronbach’s *α* coefficient for the PSS-10 was 0.81.

#### 2.2.4 Assessment of self-hate

The Self-hate Scale (SHS) (Turnell et al., 2019) is used to measure the degree of self-hate in participants. The scale consists of 7 items, scored from 1 (Strongly Disagree) to 7 (Strongly Agree). Higher scores indicate a higher degree of self-hate. The Chinese version of the SHS, revised for Chinese adolescent populations, has been proven to have excellent structural validity (confirmatory factor analysis fitting index: CFI = 0.98, TLI = 0.97, RMSEA = 0.06) and internal consistency, with a Cronbach’s α coefficient of 0.93 ~ 0.96 in previous studies (Cheng et al., 2024; Cheng et al., 2025). In this study, the internal consistency coefficient for this scale was 0.95.

#### 2.2.5 Assessment of depressive and anxiety symptoms

The Patient Health Questionnaire 4 (PHQ-4) (Wang et al., 2023) is a brief self-report measure consisting of PHQ-2 (depression) and GAD-2 (anxiety). It contains four items that assess the past 2 weeks’ emotional state, using a 4-point Likert scale (0 = “not at all,” 1 = “several days,” 2 = “more than half the days,” 3 = “nearly every day”). Higher scores reflect more severe depressive and anxiety symptoms. The Chinese version of the PHQ-4 has been verified to have good criterion validity and structural validity in Chinese adolescent and general hospital populations, with a Cronbach’s *α* coefficient of 0.85 ~ 0.90 in previous studies (Wang et al., 2023; Tan et al., 2024). The Cronbach’s α coefficient for the PHQ-4 in this study was 0.87.

### 2.3 Statistical methods

The Kolmogorov–Smirnov test and histograms were used to assess the normality of the data distribution. Data analysis was performed using SPSS 26.0 statistical software. For non-normally distributed continuous variables, median (interquartile range) [M (p25, p75)] was used to present the data. Group comparisons were performed using the Mann–Whitney *U* test and Kruskal–Wallis *H* test. Spearman correlation analysis was used to examine the relationships between variables. To ensure the rigor of the findings, common method bias was assessed using Harman’s single-factor test. Additionally, multicollinearity among the independent and mediating variables was checked by calculating the Variance Inflation Factor (VIF) and tolerance values before conducting the path analysis. To explore the relationships among ACEs, perceived stress, self-hate, and depressive and anxiety symptoms, ACEs were treated as the independent variable, perceived stress and self-hate as mediators, and depressive and anxiety symptoms as the dependent variable. The significance of the mediation effect was tested using a non-parametric percentile bootstrap method with bias correction, implemented through the SPSS PROCESS macro (Model 6). A sample size of 5,000 and a 95% confidence interval excluding 0 were considered indicative of a significant mediation effect. A *p*-value < 0.05 was considered statistically significant.

## 3 Results

### 3.1 Common method bias

A Harman’s single-factor experiment was conducted to test for the existence of common method bias, and exploratory factor analysis was performed on the data collected in this study. The results showed that a total of 6 factors with eigenvalues greater than 1 were obtained, and the variance explained by the largest factor was 31.42%, which was below the threshold of 40%. Therefore, there was no significant common method bias.

### 3.2 Statistical differences

Significant differences in ACE scores were observed based on gender, grade, whether the participant was an only child, and academic performance. Specifically, high school students (*Z* = −2.062, *p* < 0.05), only children (*Z =* −2.333, *p* < 0.05), and those with poor academic performance (*H* = 148.814, *p* < 0.05) reported higher ACE scores. Significant differences in perceived stress scores were found based on gender, grade, and academic performance. Female adolescents (*Z* = −11.293, *p* < 0.05), middle school students (*Z* = −6.575, *p* < 0.05), and those with poor or excellent academic performance (*H* = 35.757, *p* < 0.05) had higher perceived stress levels. Significant differences in self-hate scores were observed based on gender, grade, and academic performance. Female adolescents (*Z* = −11.004, *p* < 0.05), high school students (*Z* = −2.177, *p* < 0.05), and those with poor academic performance (*H* = 261.096, *p* < 0.05) had higher levels of self-hate. Significant differences in depressive and anxiety symptoms were found based on gender and academic performance. Female adolescents (*Z* = −12.731, *p* < 0.05) and those with poor academic performance (*H* = 248.119, *p* < 0.05) exhibited higher levels of depressive and anxiety symptoms (see Table 1).

**Table 1:** ACE, PSS, SHS, PHQ-4 scores in different characteristic adolescents (*n* = 7,158).

Variables	Gender		*Z*	*P*	Grade		*Z*	*P*	Only child		*Z*	*P*	Academic record			*H*	*P*
	Male	Female			Junior high school	Senior high school			Yes	No			Poor	Medium	Excellent		
	M(P25, P75)	M(P25, P75)			M(P25, P75)	M(P25, P75)			M(P25, P75)	M(P25, P75)			M(P25, P75)	M(P25, P75)	M(P25, P75)		
ACE	0(0,2)	0(0,2)	−2.020	0.043	0(0,2)	1(0,2)	−2.062	0.039	1(0,2)	0(0,2)	−2.333	0.020	1(0,3)	0(0,2)	0(0,2)	148.814	0.000
PSS	10(4,15)	12(8,16)	−11.293	0.000	12(7,16)	10(3,14)	−6.575	0.000	11(6,16)	11(7,16)	−0.585	0.558	12(7,16)	11(6,15)	12(7,16)	35.757	0.000
SHS	1(0,8)	4(0,12)	−11.004	0.000	3(0,10)	3(0,14)	−2.177	0.029	3(0,10)	3(0,10)	−0.257	0.797	7(0,15)	2(0,9)	1(0,7)	261.096	0.000
PHQ-4	1(0,3)	2(0,4)	−12.731	0.000	2(0,4)	2(0,4)	−1.323	0.186	2(0,4)	2(0,4)	−0.478	0.633	3(1,4)	2(0,4)	1(0,3)	248.119	0.000

M (P25, P75), Median and interquartile range (25th percentile, p25, and 75th percentile, p75).

ACE, Adverse Childhood Experience Scale; PSS, Perceived Stress Scale; SHS, Self-Hate Scale; PHQ-4, Patient Health Questionnaire-4.

### 3.3 Correlation analysis

Table 2 presents the correlation analysis among Adverse Childhood Experiences (ACEs), total perceived stress scores and their dimensions, self-hate, and depressive and anxiety symptoms. The analysis revealed significant positive correlations between depressive and anxiety symptoms and ACEs, total perceived stress, objective stress perception, and self-hate. Conversely, coping capacity was negatively correlated with ACEs and self-hate. Notably, all variables were significantly correlated, indicating the suitability of further testing for mediation effects.

**Table 2:** Correlation analysis between ACE, PSS, SHS and PHQ-4.

Variable	ACE	PSS-total	PSS-Stress Perception	PSS-Coping Capacity	SHS	PHQ-4
ACE	1.000					
PSS-total	0.285**	1.000				
PSS-Stress Perception	0.430**	0.803**	1.000			
PSS-Coping Capacity	−0.504**	0.692**	0.231**	1.000		
SHS	0.458**	0.380**	0.578**	−0.032**	1.000	
PHQ-4	0.428**	0.528**	0.740**	0.050**	0.565**	1.000

**p < 0.01, Spearman correlation analysis was performed.

### 3.4 Analysis of the mediating effect

Multicollinearity diagnostics showed that the VIF values for ACEs, perceived stress, and self-hate ranged from 1.32 to 2.15, all of which were below the threshold of 5. The tolerance values ranged from 0.46 to 0.76, all exceeding 0.1. These results indicate that there was no significant multicollinearity between the predictors, supporting the interpretability of the mediation model.

**Figure 1:**
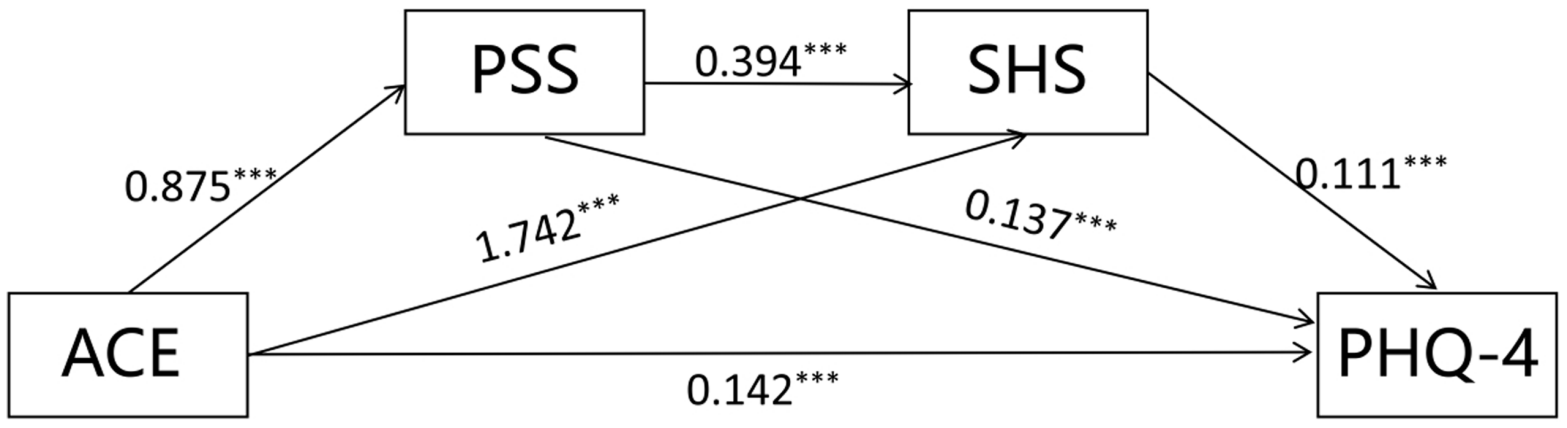
Chain mediation model between adverse childhood experiences and depression and anxiety in adolescent ACE, Adverse Childhood Experience Scale; PSS, Perceived Stress Scale; SHS, Self-hate Scale; PHQ-4, Patient Health Questionnaire-4. ***p < 0.001. Arrows represent standardized path coefficients and only represent the correlation between variables and do not indicate the direction of causal relationships. The total indirect effect (0.351) is significantly driven by three paths: ACE → PSS → PHQ-4 (24.34% of total effect), ACE → SHS PHQ-4 (39.15%), and the serial path ACE → PSS → SHS → PHQ-4 (7.71%).

The study demonstrated that perceived stress and self-hate acted as mediators in the relationship between ACEs and depressive and anxiety symptoms, with a chain mediation effect. ACEs were treated as the independent variable, PHQ-4 (depression and anxiety symptoms) as the dependent variable, and perceived stress and self-hate as mediating variables. The results indicated that the model fitted well, as shown in Figure 1. ACEs significantly predicted PHQ-4 both directly (*β* = 0.142, *p* < 0.001) and indirectly through three specific pathways: (1) via PSS alone (effect = 0.120, 95% CI: 0.106–0.135), (2) via SHS alone (effect = 0.193, 95% CI: 0.175–0.213), and (3) via the PSS-SHS chain (effect = 0.038, 95% CI: 0.033–0.044). As shown in Table 3 and visualized through the significant paths in Figure 1, the total indirect effect reached 0.351, confirming that the psychological impact of ACEs is primarily transmitted through these mediating factors.

**Table 3:** Chain mediating effects of ACE, PSS, SHS and PHQ-4.

Path	Effect	SE	95% CI	Effect size (%)
Total effect	0.493	0.013	0.468 ~ 0.518	–
Direct effect	0.142	0.011	0.119 ~ 0.164	28.80%
Indirect effect	0.351	0.014	0.324 ~ 0.380	71.20%
ACE-PSS-PHQ-4	0.120	0.007	0.106 ~ 0.135	24.34%
ACE-SHS-PHQ-4	0.193	0.010	0.175 ~ 0.213	39.15%
ACE-PSS-SHS-PHQ-4	0.038	0.003	0.033 ~ 0.044	7.71%

SE, standard error. 95% CI, 95% Confidence Interval.

## 4 Discussion

### 4.1 Differences in variable scores among adolescents with different demographic characteristics

We found that female adolescents, students reporting lower academic performance, and high school students all scored higher in ACEs, perceived stress, self-hate, and emotional distress. Only children also reported higher ACE scores than non-only children. The increased vulnerability seen in females may be due to a combination of biological and social factors. Hormonal changes, for example, can increase their stress responses (Shworth et al., 2023). Adolescents with lower academic performance often experience a harmful cycle. Garcia and Serra (2019) conducted a longitudinal study on 1,210 Spanish adolescents aged 12–16, confirming a bidirectional negative cycle between poor academic performance and emotional instability (Garcia and Serra, 2019); this is highly consistent with our findings, reflecting a universal developmental issue across cultural backgrounds. Carballo et al. (2020) conducted a meta-analysis on 18,542 European adolescents aged 11–19, showing that high school students faced more psychosocial risk factors for emotional problems (Carballo et al., 2020). Our study verifies this in the Chinese context, as Chinese high school students bear additional intense college entrance examination pressure. This suggests that entering high school is a key time for preventive interventions. The research found that the ACE scores reported by only children were higher, which was consistent with the results of Yao (2022). Yao pointed out that the total ACE score and the scores of each dimension of only children were significantly higher than those of non-only children (Yao, 2022). Although we cannot determine the specific types of abuse, our research results indicate that only children may face a higher overall burden. This may be due to the lack of opportunities for conflict resolution with siblings. The triadic family structure (two parents, one child) can become overly intense, leaving the child with no “breathing space” or alternative support when domestic stressors arise. However, Zhong et al. (2023) studied 1,521 Shanghai adolescents aged 12–18 and found no significant ACE score difference between only and non-only children; this inconsistency is mainly due to more high-quality parenting resources in Shanghai and different research age groups (Zhong et al., 2023). This tells us the link between family structure and childhood adversity is not simple. Factors like family income, how parents divide their attention and resources, and even the specific questionnaire used (e.g., CTQ vs. ACEs scale) can change this relationship (Campbell et al., 2023).

### 4.2 The impact of adverse childhood experiences on adolescent depression and anxiety

This study confirms that the cumulative burden of adverse childhood experiences (ACEs) is an important factor contributing to depression and anxiety in adolescents, which is consistent with many previous studies. Lee et al. (2020) conducted a cross-sectional study on 1,345 South Korean adolescents aged 13–18, finding ACE-exposed adolescents had a 3.2-fold higher depression risk and 2.8-fold higher anxiety risk (Lee et al., 2020); Hughes et al. (2017) completed a meta-analysis of 153,897 participants from 21 countries, confirming a dose–response relationship between ACE quantity and emotional symptom severity. Our study verifies this universal conclusion, indicating that the total accumulation of multiple childhood stressors has a harmful effect on adolescent emotional health. According to the cumulative risk perspective, it is the ACEs that lead to developmental and emotional problems (Balistreri and Alvira-Hammond, 2016). Additionally, the cumulative exposure to ACEs is known to overactivate the amygdala and slow the development of the prefrontal cortex, which weakens emotion regulation and directly contributes to symptoms of depression and anxiety (Collings and Valjee, 2024; Cross et al., 2017).

### 4.3 The mediating role of perceived stress between adverse childhood experiences and depression/anxiety

This study identified perceived stress as a mediator in the relationship between adverse childhood experiences (ACEs) and emotional distress. This result supports the “stress sensitization” theory. Yıldız and Yüksel (2024) studied 428 Turkish adolescents aged 15–18, confirming perceived stress mediated the ACEs-emotional symptoms relationship with a 22.7% effect size; our study found a 24.34% mediation effect size in Chinese adolescents, highly consistent with the above, verifying the cross-cultural commonness of this psychological mechanism. As outlined by McLaughlin et al. (2010), early adversity can trigger adaptive changes in neurobiological and cognitive-affective systems, which heighten an individual’s psychological and physiological reactivity to later stressors. Specifically, childhood trauma may dysregulate the hypothalamic–pituitary–adrenal (HPA) axis, resulting in an exaggerated and prolonged stress response—thereby increasing stress sensitivity (Yıldız and Yüksel, 2024). Furthermore, adolescents are particularly sensitive to social-evaluative stressors, such as academic evaluation and peer comparison. Within the stress sensitization framework, individuals with ACEs may show exaggerated physiological responses to these stressors, including heightened cortisol secretion and increased autonomic nervous system activation (Wendel et al., 2022). This sustained physiological reactivity may bias individuals toward perceiving everyday social situations as more threatening, thereby elevating perceived stress levels.

In our sample of Chinese adolescents, those with more ACEs reported notably higher levels of perceived stress, which in turn correlated strongly with more severe symptoms of depression and anxiety. Mechanistically, this effect may originate from the lasting influence of ACEs on the limbic-prefrontal circuit, predisposing individuals to interpret environmental cues as threatening (Teicher et al., 2016). Cognitively, early negative experiences can foster maladaptive schemas that undermine one’s sense of coping efficacy. This aligns with the negative correlation observed in this study between ACEs and the “Coping Capacity” subscale of the Perceived Stress Scale (Manyema et al., 2018; Zhao et al., 2023; O'Brien et al., 2023). High levels of perceived stress deplete adolescents’ attentional and emotion-regulatory resources, rendering them more susceptible to persistent anxiety and worry, which directly aggravate depressive and anxious symptoms (Harkness et al., 2015; Rek et al., 2022).

### 4.4 The mediating role of self-hate between adverse childhood experiences and negative emotions

This study also found that self-hate acts as a mediator between ACEs and depression and anxiety. This result supports the view proposed by Gilbert et al. (2010). Gilbert et al. (2010) studied 206 British clinical patients, finding self-hate was a core mediator between childhood trauma and emotional symptoms; Cheng et al. (2024) studied 926 Chinese adolescents aged 12–18, finding self-disgust mediated the ACEs-emotional symptoms relationship with a 38.9% effect size; our study found a 39.15% mediation effect size, almost identical to Cheng et al. (2024) and consistent with Gilbert et al. (2010), indicating self-hate is a cross-cultural core cognitive mediator. They suggested that traumatic experiences can be internalized. This internalization leads to stable negative self-evaluations and emotions, which we call self-hate. Self-hate is a core risk factor in cognitive models of depression and anxiety. Its formation is closely tied to how children explain negative events. After experiencing adversity, a child might think the bad treatment happened because of their own “flaws” or “deficits.” It solidifies into a core schema of self-criticism. Once self-hate is established, adolescents start to negatively judge their own worth, abilities, and existence (Sack et al., 2024; Yang et al., 2024). These negative judgments create feelings of helplessness, frustration, and a desire to withdraw from others. These feelings, in turn, directly contribute to anxiety and depression (Kotera et al., 2021; Cheng et al., 2024). The significant mediating role of self-hate found here is important. It highlights a key target for intervention. For adolescents with high ACEs, psychological support must actively address these distorted self-views and troubled relationships with themselves.

### 4.5 The chain mediating role of perceived stress and self-hate between adverse childhood experiences and depression and anxiety

This study identifies a chain mediation pathway where perceived stress and self-hate jointly mediate the link between ACEs and depression/anxiety. This finding supplements the single mediation mechanism and aligns with the core view of Clarke et al.’s (2019) systematic review: perceived stress triggers negative self-evaluation and worsens self-hate. This model connects stress theory with cognitive theory. It illustrates a complex, multi-stage psychological process. First, ACEs cause prolonged activation of the neuroendocrine system. This makes adolescents overly alert to everyday events. It also intensifies their emotional reactions (Jimenez et al., 2021). Next, perceived stress activates cognitive appraisal mechanisms. Individuals start to blame themselves for the stress. They may see it as proof of their own “incompetence” or “unworthiness,” rather than blaming outside circumstances. This reinforces negative self-views and promotes self-hate (Clarke et al., 2019). Then, self-hate directly results in low mood, helplessness, and ongoing anxiety. These are key psychological factors that lead to depression and anxiety.

Therefore, this research reveals a step-by-step path: ACEs increase perceived stress, and this heightened stress then worsens self-hate, which finally contributes to emotional symptoms. Nevertheless, it is important to note that this “step-by-step” pathway reflects a statistical modeling framework rather than a definitive causal sequence. In real-world contexts, perceived stress, self-hate, and emotional symptoms may interact in a recursive manner, forming a dynamic feedback loop in which each component can reinforce the others over time. Future longitudinal studies are needed to disentangle these potentially bidirectional processes.

### 4.6 Limitations and future directions

This study has meaningful findings, but it also has limitations. First, it is a cross-sectional study. This means we cannot determine the causal relationships between the variables. Our mediation model suggests possible directions of influence. However, it cannot confirm cause and effect or the order in which things happen. Future studies should use longitudinal or experimental designs. Second, all measures in this study were based on self-reports. Self-reported data can be affected by biases. For example, participants might give answers they think are socially acceptable. Future research could use more objective methods. These could include behavioral observations, physiological measurements, or clinical interviews. Using multiple methods would make the results more reliable. Third, our data came from a single source. All participants were from one region in China. Therefore, we must be careful about applying these findings to other groups. The results may not generalize to other cultures or to clinical populations. Future studies should collect data from larger and more diverse samples. These samples should include people from different regions and countries. Finally, while our large sample size (*N* = 7,158) ensures strong representativeness, the results merit cautious interpretation. In such a large cohort, even modest path coefficients can reach statistical significance (*p* < 0.001). For example, the chain mediation effect accounted for 7.71% of the total effect. This suggests that while the sequential mechanism is statistically robust, it is only one of several contributing pathways. ACEs likely influence emotional outcomes through other unmeasured routes, such as neuroendocrine dysregulation or social support. Therefore, future research should adopt a broader biopsychosocial perspective, incorporating a wider range of variables to fully capture the complex mechanisms behind adolescent emotional distress.

## 5 Conclusion

In conclusion, childhood adverse experiences can directly affect depressive-anxiety emotions, as well as indirectly mediate through perceived stress and self-hate. This study emphasizes the importance of creating a positive and healthy environment during the early stages of adolescent development, aiming to reduce childhood adverse experiences at the forefront. Additionally, it provides a theoretical guide for the early identification and treatment of depressive and anxious emotions in adolescents, thereby enhancing adolescent well-being.

## Data Availability

The original contributions presented in the study are included in the article/supplementary material, further inquiries can be directed to the corresponding authors.
